# Supplementary material for: Microenvironmental IL-6 inhibits anti-cancer immune responses generated by cytotoxic chemotherapy
Source: Nat Commun. 2021 Oct 28;12:6218. doi: 10.1038/s41467-021-26407-4 (PMC8553783; doi:10.1038/s41467-021-26407-4)
Supplement: Supplementary file 1 — Supplementary Information [file 41467_2021_26407_MOESM1_ESM.pdf]

Supplementary information

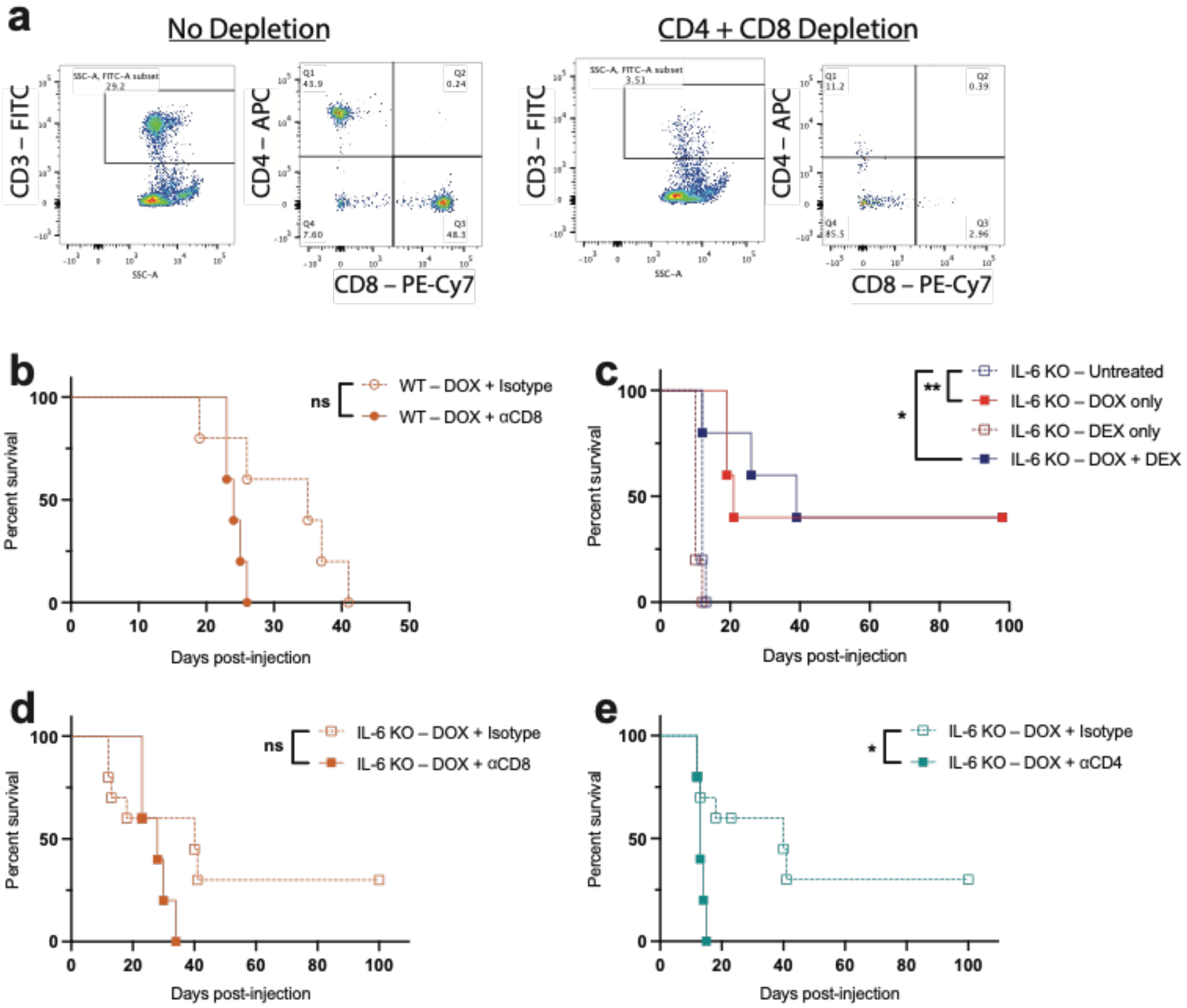

Supplementary Figure 1 | Doxorubicin and CD8/CD4 T-cell depletion treatments in WT and IL-6 KO mice. a, Flow cytometry analysis of peripheral blood samples from isotype control injected or T-cell depleted mice to confirm the efficacy of depletion. **b,** A Kaplan-Meier survival curve showing leukemic WT mice, treated with DOX and either $CD8^+$ T-cell depleted or not-depleted. n = 5 per cohort. **c,** A Kaplan-Meier survival curve showing leukemic IL-6 KO mice, treated with DOX and/or dexamethasone (DEX). n = 5 per cohort. *p = 0.0128 between IL-6 KO-untreated and IL-6 KO-DOX + DEX, **p = 0.0016 between IL-6 KO-untreated and IL-6 KO-DOX only. **d-e,** A Kaplan-Meier survival curve showing leukemic IL-6 KO mice, treated with DOX and either $CD8^+$ T-cell depleted, $CD4^+$ T-cell depleted, or not-depleted. n = 5 per cohort, except n = 10 for IL-6 KO-isotype. *p = 0.0249 in panel (**e**). Log-rank (Mantel-Cox) tests were used to compare Kaplan-Meier survival curves. Source data are provided as a 'Source Data' file.

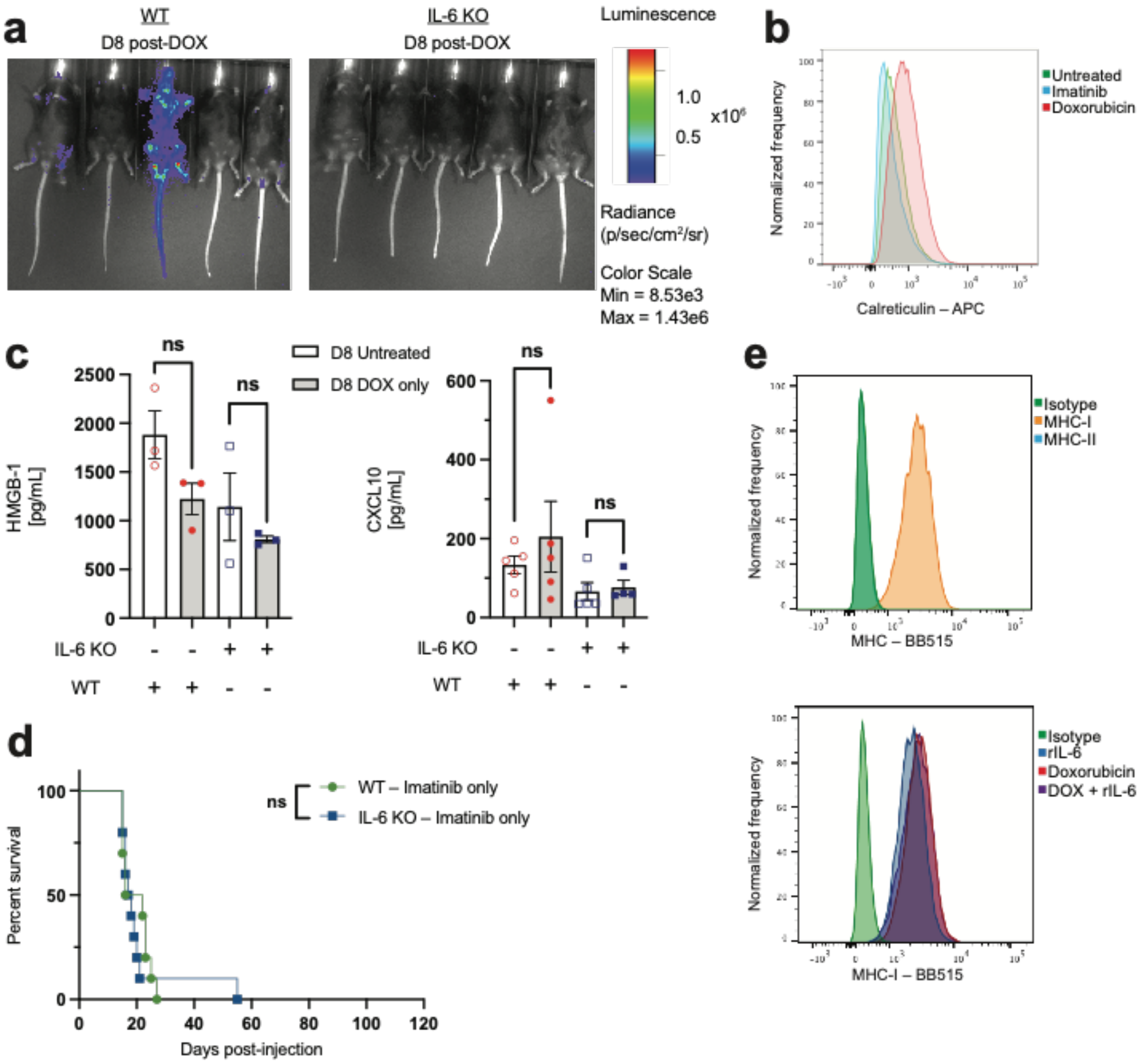

a
WT
D8 post-DOX
IL-6 KO
D8 post-DOX
Luminescence
1.0
0.5
x10⁶
Radiance
(p/sec/cm²/sr)
Color Scale
Min = 8.53e3
Max = 1.43e6
b
Normalized frequency
Untreated
Imatinib
Doxorubicin
Calreticulin – APC
c
HMGB-1 [pg/mL]
CXCL10 [pg/mL]
ns
D8 Untreated
D8 DOX only
IL-6 KO
WT
d
Percent survival
Days post-injection
WT – Imatinib only
IL-6 KO – Imatinib only
e
Isotype
MHC-I
MHC-II
MHC – BB515
rIL-6
Doxorubicin
DOX + rIL-6
MHC-I – BB515

Supplementary Figure 2 | Doxorubicin treatments and characterization of immunogenic cell death markers in B-ALL bearing WT and IL-6 KO mice. a, IVIS images showing WT or IL-6 KO mice with leukemia burden, monitored by bioluminescent imaging. **b,** A flow cytometry plot showing calreticulin expression on the surface of leukemia cells cultured *in vitro*. Cells were treated with doses that induce similar killing between both drugs and stained 18 hours after treatment initiation. **c,** Left, a graph showing the concentration of HMGB-1 present in the bone marrow of B-ALL bearing mice. n = 3 per cohort. Right, a graph showing the concentration of CXCL10 in the bone marrow of B-ALL bearing mice. n = 5 per cohort, except n = 4 for IL-6 KO-DOX only. Data is represented as mean ± SEM. Shown Ns are biological replicates from 1 independent experiment. ns>0.05 by Ordinary one-way ANOVA test. **d,** A Kaplan-Meier survival curve showing leukemic WT or IL-6 KO mice, either treated with imatinib or untreated. n = 10 per cohort. Data from 2 independent experiments is shown. Log-rank (Mantel-Cox) test. **e,** Above, a flow cytometry plot showing surface expression of MHC class I and II on leukemia cells pre-treatment *in vitro*. Histograms for MHC-II and isotype control-stained cells are overlaid. Below, a flow cytometry plot showing surface expression of MHC class I on leukemia cells after treatment with doxorubicin and/or rIL-6, *in vitro*. Data is representative of 2 independent experiments with 1 replicate per group, per experiment. D8 = Day 8. Source data are provided as a 'Source Data' file.

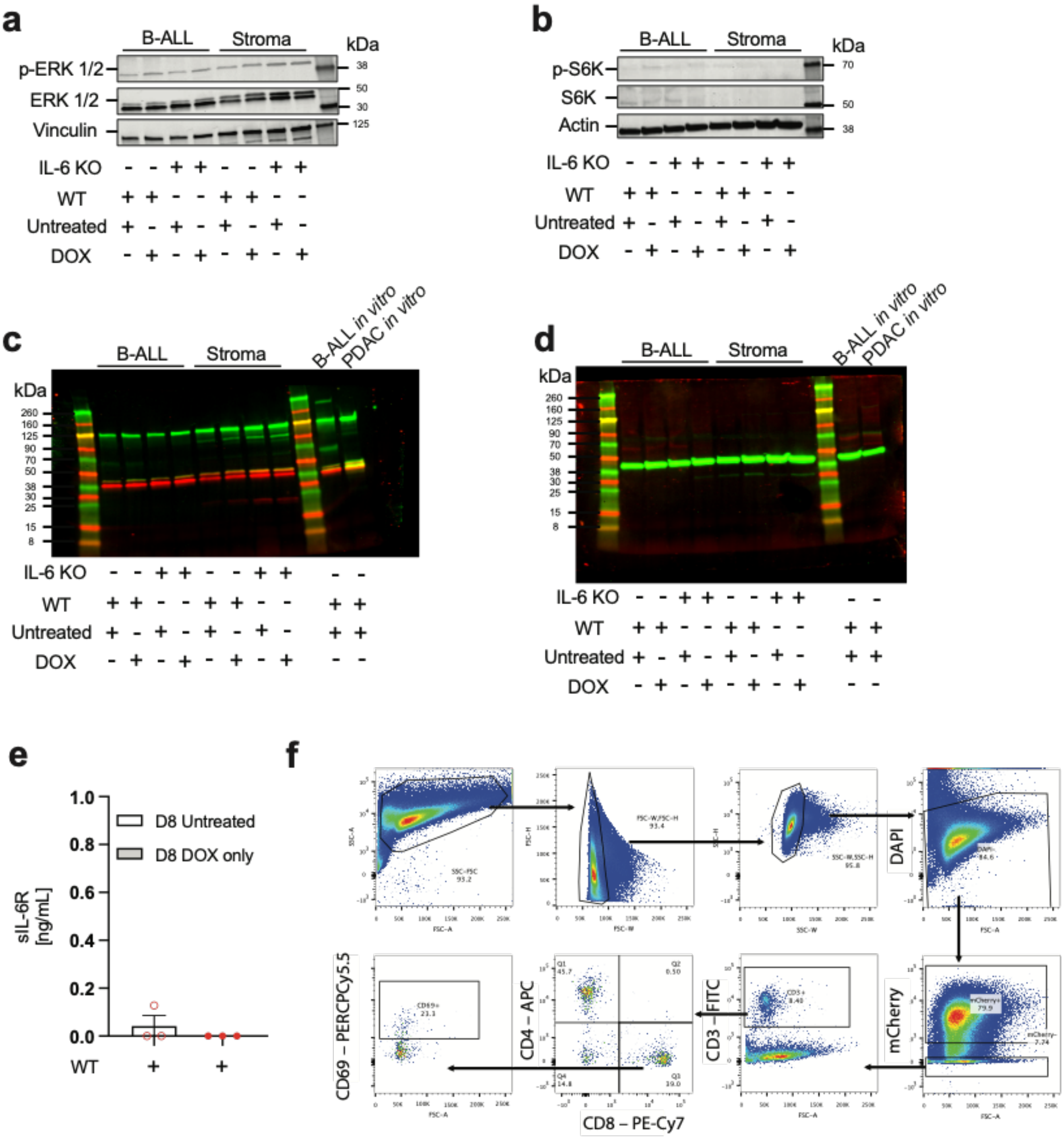
a
B-ALL
Stroma
kDa
p-ERK 1/2
ERK 1/2
Vinculin
38
50
30
125
IL-6 KO - - + + - - + +
WT + + - - + + - -
Untreated + - + - + - + -
DOX - + - + - + - +
b
B-ALL
Stroma
kDa
p-S6K
S6K
Actin
70
50
38
IL-6 KO - - + + - - + +
WT + + - - + + - -
Untreated + - + - + - + -
DOX - + - + - + - +
c
B-ALL
Stroma
B-ALL in vitro
PDAC in vitro
kDa
260
160
125
90
70
50
38
30
25
15
8
IL-6 KO - - + + - - + + - -
WT + + - - + + - - + +
Untreated + - + - + - + - + +
DOX - + - + - + - + - -
d
B-ALL
Stroma
B-ALL in vitro
PDAC in vitro
kDa
260
160
125
90
70
50
38
30
25
15
8
IL-6 KO - - + + - - + + - -
WT + + - - + + - - + +
Untreated + - + - + - + - + +
DOX - + - + - + - + - -
e
sIL-6R [ng/mL]
1.0
0.8
0.6
0.4
0.2
0.0
D8 Untreated
D8 DOX only
WT + +
f
DAPI
mCherry
CD3 – FITC
CD4 – APC
CD69 – PERCPCy5.5
CD8 – PE-Cy7

Supplementary Figure 3 | IL-6 downstream signaling characterization and identification of immune infiltrates. a, ERK 1/2 activity as assessed by Western blot analysis 24 hours after DOX treatment in B-ALL and stroma samples from WT and IL-6 KO mice. **b,** S6K activity as assessed by Western blot analysis 24 hours after DOX treatment in B-ALL and stroma samples from WT and IL-6 KO mice. Representative blots of 4 biological replicates per sample for both (**a**) and (**b**). **c-d,** Full overlayed Western blots with marks of protein size for blots shown in (**a**) and (**b**), respectively. **e,** A graph showing the concentration of sIL-6R present in the bone marrow of WT B-ALL bearing mice. Data is represented as mean ± SEM. Shown Ns are biological replicates from 1 independent experiment. **f,** Flow cytometry analysis for the identification of T-cell subsets. Source data are provided as a 'Source Data' file.

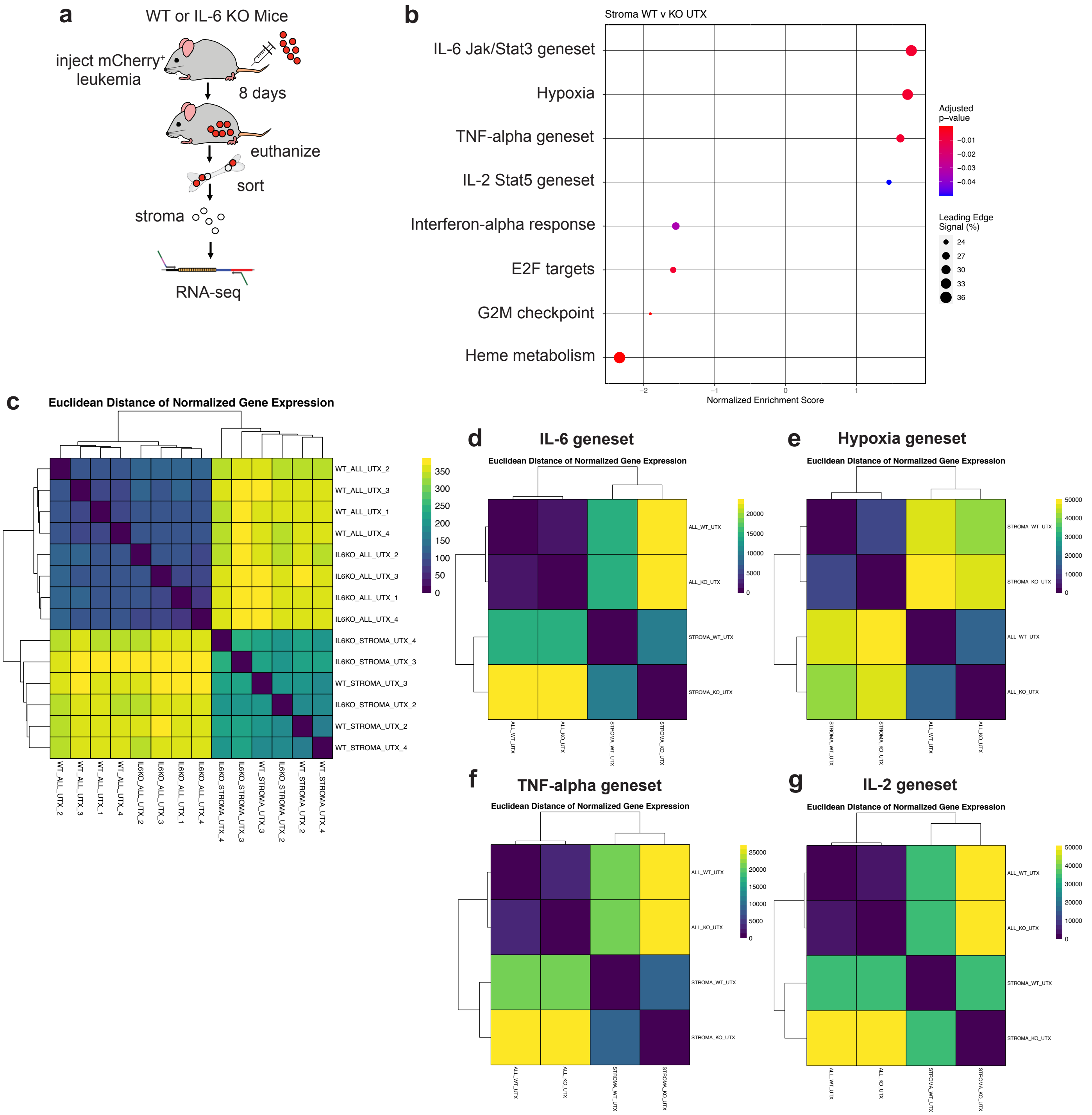
a
WT or IL-6 KO Mice
inject mCherry+ leukemia
8 days
euthanize
sort
stroma
RNA-seq
b
Stroma WT v KO UTX
IL-6 Jak/Stat3 geneset
Hypoxia
TNF-alpha geneset
IL-2 Stat5 geneset
Interferon-alpha response
E2F targets
G2M checkpoint
Heme metabolism
Normalized Enrichment Score
Adjusted p-value
Leading Edge Signal (%)
c
Euclidean Distance of Normalized Gene Expression
WT_ALL_UTX_2
WT_ALL_UTX_3
WT_ALL_UTX_1
WT_ALL_UTX_4
IL6KO_ALL_UTX_2
IL6KO_ALL_UTX_3
IL6KO_ALL_UTX_1
IL6KO_ALL_UTX_4
IL6KO_STROMA_UTX_4
IL6KO_STROMA_UTX_3
WT_STROMA_UTX_3
IL6KO_STROMA_UTX_2
WT_STROMA_UTX_2
WT_STROMA_UTX_4
d
IL-6 geneset
Euclidean Distance of Normalized Gene Expression
e
Hypoxia geneset
Euclidean Distance of Normalized Gene Expression
f
TNF-alpha geneset
Euclidean Distance of Normalized Gene Expression
g
IL-2 geneset
Euclidean Distance of Normalized Gene Expression
ALL_WT_UTX
ALL_KO_UTX
STROMA_WT_UTX
STROMA_KO_UTX

Supplementary Figure 4 | RNA-sequencing from bone marrow. a, Schematic outline of experiment to isolate bone marrow stromal and leukemia cells from WT and IL-6 KO mice for RNA-sequencing. 500,000 cells per mouse were injected and mice sacked 8 days later, bone marrow collected and $mCherry^{+}$ leukemia cells, and $mCherry^{-}$ stromal cells, sorted and RNA isolated for RNA-sequencing. **b,** GSEA analysis of differential gene expression in stroma from IL-6 KO relative to wild-type samples using the MSigDB GO cancer Hallmarks collection. All significant results (adjusted p-value<0.05) are included and sorted by normalized enrichment score, and rank-listed by t-statistic. Dot size corresponds to the fraction of the gene set in the leading-edge signal. Color corresponds to adjusted p-value. **c,** Euclidean distance between global normalized gene expression. **d-g,** Euclidean distance between mean sample gene expression for enriched gene sets within the GSEA GO Hallmarks collection.

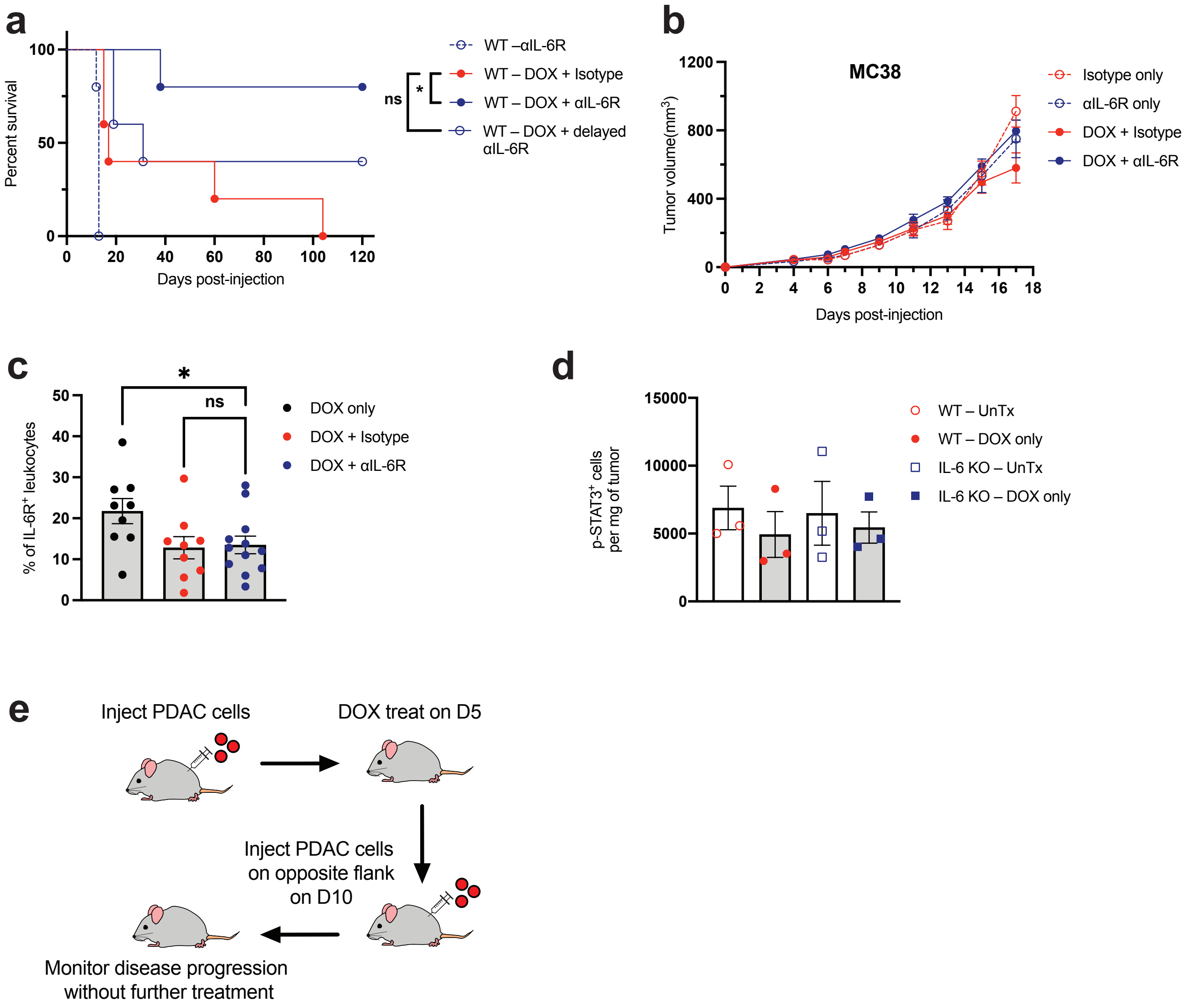
a
Percent survival
Days post-injection
WT –αIL-6R
WT – DOX + Isotype
WT – DOX + αIL-6R
WT – DOX + delayed αIL-6R
ns
*
b
MC38
Tumor volume(mm3)
Days post-injection
Isotype only
αIL-6R only
DOX + Isotype
DOX + αIL-6R
c
% of IL-6R+ leukocytes
DOX only
DOX + Isotype
DOX + αIL-6R
d
p-STAT3+ cells per mg of tumor
WT – UnTx
WT – DOX only
IL-6 KO – UnTx
IL-6 KO – DOX only
e
Inject PDAC cells
DOX treat on D5
Inject PDAC cells on opposite flank on D10
Monitor disease progression without further treatment

Supplementary Figure 5 | αIL-6R treatment optimization. a, A Kaplan-Meier survival curve showing WT mice that underwent treatment optimization with therapeutic antibodies against IL-6R. Treatment for all groups started on Day 3 after leukemia transplantation and continued for every other day. Mice on the 'DOX + delayed αIL-6R' group received inhibitor treatment starting on Day 7 after leukemia transplant and continued for every other day. n = 5 per cohort. *p = 0.0126 by Log-rank (Mantel-Cox) test. **b,** A graph showing MC38 tumor burden in WT mice treated with DOX and either an IL-6R blocking antibody or isotype control. n = 5 per cohort. Data is represented as mean ± SEM. **c,** A graph showing IL-6R expression percentages in the dissociated PDAC tumors from the indicated treatment groups. Data was quantified by flow cytometry, and is represented as mean ± SEM. n = 9 per cohort, except n = 12 for DOX + αIL-6R. *p = 0.0339 by two-tailed Student t-test. **d,** A graph showing the total number of p-STAT3 positive cells per mg of PDAC tumor in the indicated treatment groups. Data was quantified by flow cytometry, and is represented as mean ± SEM. n = 3 per cohort. **e,** Schematic outline of IL-6 KO mice previously treated with DOX, re-transplanted with PDAC cells, and then tumor burden progression monitored in the absence of further treatment. Data in panels (**b-d**) is represented as mean ± SEM. D5 = Day 5, D10 = Day 10. Source data are provided as a 'Source Data' file.

Table BM	Untreated		D2 post-DOX	
**Immune Cell Population in BM (Percent of mCherry- cells)**	**WT**	**IL-6 KO**	**WT**	**IL-6 KO**
**T Cells**				
1) CD3+	3.36	2.94	7.84[a]	8.10[b]
2) CD3+ CD4+	1.80	1.42	3.22[c]	3.40[d]
3) CD3+ CD4+ CD69+	0.41	0.28	0.67	0.58[e]
4) CD3+ CD4+ CD25+	0.15	0.11	0.13	0.19[f]
5) CD3+ CD8+	0.40	0.50	2.62[g]	3.18[h]
6) CD3+ CD8+ CD69+	0.30	0.33	0.87[i]	0.93[j]
**Dendritic Cells**				
7) CD11c+ MHCII+	1.80	1.70	3.43	3.21[k]
8) CD11c+ MHCII+ CD11b+	1.27	1.05	2.24[l]	1.88
9) CD11c+ MHCII+ CD11b+ CD86+	0.51	0.46	0.77	0.67
10) CD11c+ MHCII+ CD11b-	0.96	0.76	1.91	1.37
11) CD11c+ MHCII+ CD11b- CD86+	0.27	0.24	0.66	0.51
12) CD11c+ MHCII+ CD11b- CD103+	0.51	0.41	0.64	0.82
13) CD11c+ MHCII+ CD11b- CD103+ CD86+	0.22	0.17	0.26	0.38
**Macrophages**				
14) F480+ CD11b+ Gr-1-	1.60	1.65	1.19[m]	1.32
15) F480+ CD11b+ Gr-1- MHCII+	0.25	0.20	0.48[n]	0.42[o]
**Neutrophils**				
16) F480- CD11b+ Gr-1+	21.31	27.90	37.67[p]	39.00[q]

Supplementary Table 1 | Quantification of bone marrow immune infiltration before and after doxorubicin treatment. The average representation of immune cell subsets in the bone marrow of WT and IL-6 KO mice before and after treatment. Superscripts denote $p<0.05$ compared to untreated sample of the same genetic background. Analyzed by two-tailed Student t-test. Superscripts corresponding exact p-values: a = 0.0142, b<0.0001, c = 0.0044, d<0.0001, e = 0.0137, f = 0.0059, g = 0.0099, h<0.0001, i = 0.0032, j = 0.0018, k = 0.019, l = 0.0061, m = 0.0071, n = 0.0271, o = 0.0176, p = 0.0029, and q = 0.0189. Samples in group 4 have: n = 7 for WT-untreated, n = 8 for WT-D2 post-doxorubicin, n = 7 for IL-6 KO-untreated, n = 8 IL-6 KO-D2 post-doxorubicin mice, and show data from 3 independent experiments. Samples in groups 1, 2, 5, 7, 14, and 16 have: n = 7 for WT-untreated, n = 11 for WT-D2 post-doxorubicin, n = 7 for IL-6 KO-untreated, n = 10 IL-6 KO-D2 post-doxorubicin mice, and show data from 4 independent experiments. Samples in groups 3, 6, 8-13, and 15 have: n = 9 for WT-untreated, n = 10 for WT-D2 post-doxorubicin, n = 9 for IL-6 KO-untreated, n = 8 IL-6 KO-D2 post-doxorubicin mice, and show data from 3 independent experiments. There were no significant statistical comparisons between 'untreated' and 'DOX treated' samples of different genetic backgrounds. Source data are provided as a 'Source Data' file.

Table Spleen	Untreated		D2 post-DOX	
**Immune Cell Population in Spleen (Percent of mCherry- cells)**	**WT**	**IL-6 KO**	**WT**	**IL-6 KO**
**T Cells**				
1) CD3+	17.30	17.42	19.18	19.34
2) CD3+ CD4+	9.74	9.08	9.85	9.53
3) CD3+ CD4+ CD69+	0.61	0.53	1.16[a]	1.05[b]
4) CD3+ CD4+ CD25+	0.54	0.51	0.58	0.67
5) CD3+ CD8+	5.52	6.32	6.90	7.59
6) CD3+ CD8+ CD69+	0.21	0.24	0.43[c]	0.49[d]
**Dendritic Cells**				
7) CD11c+ MHCII+	2.26	1.86	5.13[e]	4.60[f]
8) CD11c+ MHCII+ CD11b+	0.96	0.81	1.34[g]	1.26[h]
9) CD11c+ MHCII+ CD11b+ CD86+	0.41	0.37	0.60[i]	0.61[j]
10) CD11c+ MHCII+ CD11b-	1.32	1.08	3.95[k]	3.09[l]
11) CD11c+ MHCII+ CD11b- CD86+	0.49	0.57	0.94[m]	1.09
12) CD11c+ MHCII+ CD11b- CD103+	0.58	0.52	1.24	1.55
13) CD11c+ MHCII+ CD11b- CD103+ CD86+	0.22	0.18	0.46	0.55
**Macrophages**				
14) F480+ CD11b+ Gr-1-	0.57	0.82	0.83[n]	0.85
15) F480+ CD11b+ Gr-1- MHCII+	0.29	0.33	0.41[o]	0.44
**Neutrophils**				
16) F480- CD11b+ Gr-1+	2.04	2.19	0.82[p]	0.81[q]

Supplementary Table 2 | Quantification of spleen immune infiltration before and after doxorubicin treatment. The average representation of immune subsets in the spleen of WT and IL-6 KO mice before and after treatment. Superscripts denote $p<0.05$ compared to untreated sample of the same genetic background. Analyzed by two-tailed Student t-test. Superscripts corresponding exact p-values: a = 0.0244, b = 0.0062, c = 0.0235, d = 0.0028, e = 0.0014, f = 0.0003, g = 0.0034, h<0.0001, i = 0.002, j = 0.001, k = 0.0232, l = 0.0182, m = 0.0208, n = 0.0289, o = 0.0132, p = 0.002, and q = 0.0118. Samples in group 1, 2, 5, and 7 have: n = 12 for WT-untreated, n = 14 for WT-D2 post-doxorubicin, n = 12 for IL-6 KO-untreated, n = 12 IL-6 KO-D2 post-doxorubicin mice, and show data from 4 independent experiments. Samples in groups 14, 15, and 16 have: n = 12 for WT-untreated, n = 13 for WT-D2 post-doxorubicin, n = 12 for IL-6 KO-untreated, n = 12 IL-6 KO-D2 post-doxorubicin mice, and show data from 4 independent experiments. Samples in groups 3, 4, 6, and 8-13 have: n = 9 for WT-untreated, n = 10 for WT-D2 post-doxorubicin, n = 9 for IL-6 KO-untreated, n = 9 IL-6 KO-D2 post-doxorubicin mice, and show data from 3 independent experiments. There were no significant statistical comparisons between 'untreated' and 'DOX treated' samples of different genetic backgrounds. Source data are provided as a 'Source Data' file.
