# Supplementary material for: Microenvironmental IL-6 inhibits anti-cancer immune responses generated by cytotoxic chemotherapy
Source: Nat Commun. 2021 Oct 28;12:6218. doi: 10.1038/s41467-021-26407-4 (PMC8553783; doi:10.1038/s41467-021-26407-4)
Supplement: Supplementary file 3 — Reporting Summary [file 41467_2021_26407_MOESM3_ESM.pdf]

nature research

Corresponding author(s): Michael T. Hemann

Last updated by author(s): Sep 27, 2021

Reporting Summary

Nature Research wishes to improve the reproducibility of the work that we publish. This form provides structure for consistency and transparency in reporting. For further information on Nature Research policies, see our Editorial Policies and the Editorial Policy Checklist.

Statistics

For all statistical analyses, confirm that the following items are present in the figure legend, table legend, main text, or Methods section.

n/a	Confirmed	
☐	☒	The exact sample size (*n*) for each experimental group/condition, given as a discrete number and unit of measurement
☐	☒	A statement on whether measurements were taken from distinct samples or whether the same sample was measured repeatedly
☐	☒	The statistical test(s) used AND whether they are one- or two-sided<br>*Only common tests should be described solely by name; describe more complex techniques in the Methods section.*
☐	☒	A description of all covariates tested
☐	☒	A description of any assumptions or corrections, such as tests of normality and adjustment for multiple comparisons
☐	☒	A full description of the statistical parameters including central tendency (e.g. means) or other basic estimates (e.g. regression coefficient) AND variation (e.g. standard deviation) or associated estimates of uncertainty (e.g. confidence intervals)
☐	☒	For null hypothesis testing, the test statistic (e.g. *F*, *t*, *r*) with confidence intervals, effect sizes, degrees of freedom and *P* value noted<br>*Give P values as exact values whenever suitable.*
☒	☐	For Bayesian analysis, information on the choice of priors and Markov chain Monte Carlo settings
☒	☐	For hierarchical and complex designs, identification of the appropriate level for tests and full reporting of outcomes
☒	☐	Estimates of effect sizes (e.g. Cohen's *d*, Pearson's *r*), indicating how they were calculated

Our web collection on statistics for biologists contains articles on many of the points above.

Software and code

Policy information about availability of computer code

Data collection	No custom software or instrument was used for data collection in this study. Data was collected with the following tools: Illumina NovaSeq 6000, BD FACSDiva, Odyssey CLx (LI-COR imaging system), and IVIS Spectrum-bioluminescence and fluorescence imaging system (Perkin Elmer).
Data analysis	No custom software or code was used for data analysis in this study. Data was analyzed with the following tools: Microsoft Excel, GraphPad Prism9, FlowJo, Salmon (version 1.3.0), DESeq2 (version 1.28.1), R (version 3.16.1), GSEA analysis, and Living Image software.

For manuscripts utilizing custom algorithms or software that are central to the research but not yet described in published literature, software must be made available to editors and reviewers. We strongly encourage code deposition in a community repository (e.g. GitHub). See the Nature Research guidelines for submitting code & software for further information.

Data

Policy information about availability of data

All manuscripts must include a data availability statement. This statement should provide the following information, where applicable:

- Accession codes, unique identifiers, or web links for publicly available datasets
- A list of figures that have associated raw data
- A description of any restrictions on data availability

The datasets and analysis generated during the current study are available as a 'Source Data' file. The gene expression datasets generated during this study is available from the National Center for Biotechnology Information Gene Expression Omnibus (GEO), and are accessible through GEO Series with the following accession number: GSE184107.

Field-specific reporting

Please select the one below that is the best fit for your research. If you are not sure, read the appropriate sections before making your selection.

☒ Life sciences ☐ Behavioural & social sciences ☐ Ecological, evolutionary & environmental sciences

For a reference copy of the document with all sections, see nature.com/documents/nr-reporting-summary-flat.pdf

Life sciences study design

All studies must disclose on these points even when the disclosure is negative.

Sample size	When possible, cohorts of 5 mice per cage were used. Key findings were repeated in multiple independent experiments, as noted in figure legends. No sample size calculation was performed. Sample sizes are chosen with the intent to have equal number of animals per group. Due to age gaps or sex differences of available mice, some experiments have slightly different sample size numbers. However, the use of slightly distinct mouse numbers does not preclude robust statistical comparisons, especially when the recorded observations remain true across independent experiments/repetitions.
Data exclusions	No data was excluded from the analysis. If present, outliers were included in the reported data.
Replication	Key findings were repeated in multiple independent experiments, as noted in figure legends.
Randomization	Randomization of animal cohorts was performed before transplantation of disease and before the start of any treatment. For other experiments using mouse harvested tissues/cells, allocation of samples was based on mice cohort conditions. For experiments using cells in vitro, sample allocation was also determined on group/treatment conditions. Key findings were repeated with independent experiments.
Blinding	When able, the experimenter was blinded to the individual mice being examined, although this was not performed in all experiments. Researchers were specifically blinded when collecting IVIS-bioluminescence data, with samples typically being handled for imaging by one scientist and data processing performed by another. Similarly, for immuno-phenotyping experiments by flow cytometry, one scientist typically processed and prepared samples, while the results were properly labeled and assigned by another.

Reporting for specific materials, systems and methods

We require information from authors about some types of materials, experimental systems and methods used in many studies. Here, indicate whether each material, system or method listed is relevant to your study. If you are not sure if a list item applies to your research, read the appropriate section before selecting a response.

Materials & experimental systems

n/a	Involved in the study	
☐	☒	Antibodies
☐	☒	Eukaryotic cell lines
☒	☐	Palaeontology and archaeology
☐	☒	Animals and other organisms
☒	☐	Human research participants
☒	☐	Clinical data
☒	☐	Dual use research of concern

Methods

n/a	Involved in the study	
☒	☐	ChIP-seq
☐	☒	Flow cytometry
☒	☐	MRI-based neuroimaging

Antibodies

Antibodies used	CD3–FITC (17A2, BioLegend #100204; 1:100), CD4–APC (RM4-5, BD Biosciences #561091; 1:100), CD4–APC-Cy7 (GK1.5, BioLegend #100414; 1:100), CD8–PE-Cy7 (53-6.7, BD Biosciences #552877; 1:100), CD25–APC-Cy7 (PC61, BioLegend #102026; 1:100), CD69–PerCP-Cy5.5 (H1.2F3, BioLegend #104522; 1:100), CD11c–FITC (HL3, BD Biosciences #553801; 1:100), CD103–PerCP-Cy5.5 (2E7, BioLegend #121416; 1:100), CD86–APC (GL-1, BioLegend #105012; 1:100), MHC-II–APC-Cy7 (M5/114.15.2, BioLegend #107628; 1:100), MHC-II–PerCP-Cy5.5 (M5/114.15.2, BioLegend #107626; 1:100), CD11b–PE-Cy7 (M1/70, BioLegend #101216; 1:100), F4/80–APC (BM8, BioLegend #123116; 1:100), Gr-1–FITC (RB6-8C5, eBioscience #50-991-9; 1:100), IL-6R–APC (D7715A7, BioLegend #115812; 1:100), PD-1–BV421 (29F.1A12, BioLegend #135217; 1:100), MHC-I–FITC (34-1-2S, Abcam #ab95572; 1:100), MHC-II–FITC (M5/114, Abcam #ab239229; 1:100), PD-L1–PE-Cy7 (10F.9G2, BioLegend #124314; 1:100), p-STAT3 (Tyr705, D3A7, Cell Signaling Technology #4323S; 1:25), IgG-isotype control (DA1E, Cell Signaling Technology #2975S; 1:25), CD3–BV605 (17A2, BioLegend #100237; 1:100), Zombie Aqua Fixable Viability Dye (BioLegend #423102; 1:100), anti-actin (13E5, Cell Signaling Technology #4970S; 1:1000), anti-S6K (R&D Systems #AF8964; 1:200), anti-p-S6K (Thr389, Cell Signaling Technology #9205S; 1:1000), anti-vinculin (E1E9V, Cell Signaling Technology #13901S; 1:1000), anti-ERK (W15133B, BioLegend #686902; 1:1000), anti-p-ERK (Thr202/Tyr204, Cell Signaling Technology #9101S; 1:1000), anti-rat IRDye 680RD (LI-COR #926-68076; 1:5000), anti-rabbit IRDye 800CW (LI-COR #926-32211; 1:5000), anti-goat IRDye 680RD (LI-COR #926-68074; 1:5000), anti-rabbit IRDye 800CW (LI-COR #926-32213; 1:5000), CD4 (GK1.5, BioXCell #BE0003-1), CD8 (2.43, BioXCell), PD-L1 (10F.9G2, BioXCell), IL-6R Ab (15A7, BioXCell), and Rat IgG2b-isotype control (LTF-2, BioXCell).

Validation

CD3–FITC (17A2, BioLegend #100204) website validation: 101 citations, splenocytes from C57BL/6 mice successfully stained with 17A2 FITC.

CD4–APC (RM4-5, BD Biosciences #561091) website validation: 14 citations, splenocytes successfully stained.

CD4–APC-Cy7 (GK1.5, BioLegend #100414) website validation: 64 citations, splenocytes from C57BL/6 mice successfully stained.

CD8–PE-Cy7 (53-6.7, BD Biosciences #552877) website validation: 24 citations, routinely tested in flow cytometry applications.

CD25–APC-Cy7 (PC61, BioLegend #102026) website validation: 23 citations, splenocytes from C57BL/6 mice successfully stained.

CD69–PerCP-Cy5.5 (H1.2F3, BioLegend #104522) website validation: 21 citations, PMA + ionomycin stimulated splenocytes from C57BL/6 mice successfully stained.

CD11c–FITC (HL3, BD Biosciences #553801) website validation: 8 citations, splenocytes successfully stained.

CD103–PerCP-Cy5.5 (2E7, BioLegend #121416) website validation: 28 citations, splenocytes from C57BL/6 mice successfully stained.

CD86–APC (GL-1, BioLegend #105012) website validation: 35 citations, LPS stimulated splenocytes from C57BL/6 mice successfully stained.

MHC-II–APC-Cy7 (M5/114.15.2, BioLegend #107628) website validation: 48 citations, splenocytes from C57BL/6 mice successfully stained.

MHC-II–PerCP-Cy5.5 (M5/114.15.2, BioLegend #107626) website validation: 36 citations, splenocytes from C57BL/6 mice successfully stained.

CD11b–PE-Cy7 (M1/70, BioLegend #101216) website validation: 156 citations, bone marrow cells from C57BL/6 mice successfully stained.

F4/80–APC (BM8, BioLegend #123116) website validation: 213 citations, thioglycolate-elicited macrophages from BALB/c mice successfully stained.

Gr-1–FITC (RB6-8C5, eBioscience #50-991-9) website validation: splenocytes and bone marrow cells successfully stained.

IL-6R–APC (D7715A7, BioLegend #115812) website validation: 4 citations, mouse myeloma FO cells successfully stained.

PD-1–BV421 (29F.1A12, BioLegend #135217) website validation: 20 citations, Con-A and IL-2 stimulated splenocytes from C57BL/6 mice successfully stained.

MHC-I–FITC (34-1-2S, Abcam #ab95572) website validation: 1 citation, splenocytes successfully stained.

MHC-II–FITC (M5/114, Abcam #ab239229) website validation: splenocytes successfully stained.

PD-L1–PE-Cy7 (10F.9G2, BioLegend #124314) website validation: 14 citations, splenocytes from C57BL/6 mice successfully stained.

p-STAT3 (Tyr705, D3A7, Cell Signaling Technology #4323S) website validation: 5 citations, Jurkat cells stimulated with IFN-alpha successfully stained.

IgG-isotype control (DA1E, Cell Signaling Technology #2975S) website validation: 22 citations, used as control in experiments with Jurkat cells stimulated with IFN-alpha.

CD3–BV605 (17A2, BioLegend #100237) website validation: 26 citations, splenocytes from C57BL/6 mice successfully stained.

Zombie Aqua Fixable Viability Dye (BioLegend #423102) website validation: 173 citations, day-old splenocytes, either fixed or unfixed, successfully stained.

anti-actin (13E5, Cell Signaling Technology #4970S) website validation: 3,054 citations, Western blot analysis of recombinant actin isoforms successfully stained.

anti-S6K (R&D Systems #AF8964) website validation: Western blot analysis of human breast cancer cell line, HeLa cells, and NIH-3T3 mouse cell line successfully stained.

anti-p-S6K (Thr389, Cell Signaling Technology #9205S) website validation: 1,218 citations, Western blot analysis of HeLa, COS, C6, and NIH-3T3 cell lines successfully stained.

anti-vinculin (E1E9V, Cell Signaling Technology #13901S) website validation: 143 citations, Western blot analysis of various cell lines successfully stained.

anti-ERK (W15133B, BioLegend #686902) website validation: 1 citation, Western blot analysis of 293T cells exposed to various conditions successfully stained.

anti-p-ERK (Thr202/Tyr204, Cell Signaling Technology #9101S) website validation: 5,715 citations, Western blot analysis of mouse embryonic fibroblasts exposed to various conditions successfully stained.

CD4 (GK1.5, BioXCell #BE0003-1) website validation: 17 citations, reported applications include CD4+ T-cell depletion.

CD8 (2.43, BioXCell #BE0061) website validation: 17 citations, reported applications include CD8+ T-cell depletion.

PD-L1 (10F.9G2, BioXCell #BE0101) website validation: 18 citations, reported applications include in vivo PD-L1 blockade.

IL-6R Ab (15A7, BioXCell #BE0047) website validation: 5 citations, reported applications include in vivo blockade of IL-6/IL-6R signaling.

Rat IgG2b-isotype control (LTF-2, BioXCell #BE0090) website validation: 15 citations, reacts with a protein not expressed my mammals making it ideal for use as an isotype control.

Eukaryotic cell lines

Policy information about cell lines

Cell line source(s)	Luciferase+ BCR-ABL+ B-ALL murine cells were a gift from Richard Williams (Proc. Natl. Acad. Sci. USA 103, 2006). PDAC murine cells were isolated from autochthonous tumors and a gift from Matthew Vander Heiden Lab. Autochthonous model was generated in the David A. Tuveson Lab. (Cancer Cell, 2005). MC38 or Colon 38 cells were acquired from the Developmental Therapeutics Program Tumor Repository at Frederick National Laboratory.
Authentication	The cell lines were not independently authenticated.
Mycoplasma contamination	Cells regularly tested negative for Mycoplasma detection (MycoAlert Plus kit, Lonza).
Commonly misidentified lines (See ICLAC register)	No commonly misidentified cell lines were used in this study.

Animals and other organisms

Policy information about studies involving animals; ARRIVE guidelines recommended for reporting animal research

Laboratory animals	C57BL/6J and C57BL/6J IL-6-/- mice, 6-8 week-old, were purchased from Jackson Laboratory and breeding colony maintained (RRID: IMSR_JAX:000664, and IMSR_JAX:002650). Both female and male sexes were used. Food (ProLab RMH 3000) and water were given ad libitum. Animals were housed at 68-72 °F, with a relative humidity of 30-70%, and a dark/light cycle of 12/12 hours.
Wild animals	This study did not involve wild animals.
Field-collected samples	This study did not involve samples collected from the field.
Ethics oversight	Koch Institute and the Massachusetts Institute of Technology Department of Comparative Medicine approved all procedures and animal handling of the study.

Note that full information on the approval of the study protocol must also be provided in the manuscript.

Flow Cytometry

Plots

Confirm that:

- [x] The axis labels state the marker and fluorochrome used (e.g. CD4-FITC).
- [x] The axis scales are clearly visible. Include numbers along axes only for bottom left plot of group (a 'group' is an analysis of identical markers).
- [x] All plots are contour plots with outliers or pseudocolor plots.
- [x] A numerical value for number of cells or percentage (with statistics) is provided.

Methodology

Sample preparation	Leukemic mice were sacrificed 8 days post-injection (untreated), 2 days after doxorubicin, or 7 days post-treatment for analysis of immune-cell infiltration in bone marrow and spleen. Bone-marrow cells from WT and IL-6 KO mice were extracted by crushing both femurs and tibias with mortar and pestle in RBC Lysing Buffer (Sigma) for 5 minutes and re-suspended in 3% FBS-PBS (FACS Stain buffer). Splenic cells were extracted by crushing the spleen between glass slides into RBC Lysing Buffer. Bone marrow and splenic cells from WT and IL-6 KO mice were extracted as described above, fixed in 3 or 4% paraformaldehyde, stained with primary p-STAT3.

Instrument	FACS-AriaIII (Becton Dickinson), and LSRII HTS flow cytometer (Becton Dickinson).
Software	BD FACSDiva Software was used for collection of the data and FlowJo Software for analysis.
Cell population abundance	Quantified as proportion of parent population, as proportion of total or absolutely with use of counting beads. mCherry+ B-ALL cells were sorted twice on a FACS-AriaIII (Becton Dickinson) to get a pure mCherry+ population.
Gating strategy	Population of interest is first identified from a FSC-A and SSC-A plot. Single cells are then gated for both height and width from SSC and FSC plots. A live/dead exclusion dye is used to identify live population of cells and then gating is performed around fluorophores/markers of interest. Single stain controls were used to identify positive from negative stained cells and to set gates and compensation.

☒ Tick this box to confirm that a figure exemplifying the gating strategy is provided in the Supplementary Information.
